# Tirbanibulin 1% Ointment for Actinic Keratosis: Results from a Real-Life Study

**DOI:** 10.3390/medicina60020225

**Published:** 2024-01-28

**Authors:** Federica Li Pomi, Mario Vaccaro, Giovanni Pallio, Michelangelo Rottura, Natasha Irrera, Francesco Borgia

**Affiliations:** 1 Department of Clinical and Experimental Medicine, Section of Dermatology, University of Messina, 98125 Messina, Italy; federicalipomi@hotmail.it (F.L.P.); mario.vaccaro@unime.it (M.V.); 2 Department of Biomedical and Dental Sciences and Morphological and Functional Imaging, University of Messina, 98125 Messina, Italy; giovanni.pallio@unime.it; 3 Department of Clinical and Experimental Medicine, Section of Pharmacology, University of Messina, 98125 Messina, Italy; michelangelo.rottura@unime.it (M.R.); natasha.irrera@unime.it (N.I.)

**Keywords:** tirbanibulin, tirbanibulin ointment, actinic keratosis, real-life

## Abstract

*Background and Objectives*: Tirbanibulin 1% ointment is a novel synthetic anti-proliferative agent that inhibits tubulin polymerization. It is approved for treating actinic keratosis (AK) on the face and scalp in adults. It has demonstrated good efficacy, an adequate safety profile and excellent patient adherence in the phase 3 clinical trials, however data about its real-life efficacy and safety are lacking. Here we report the experience of the dermatology unit of the University Hospital of Messina. *Materials and Methods*: We performed a spontaneous open-label, prospective non-randomized study to assess the effectiveness and safety of tirbanibulin 1% ointment for the treatment of 228 AKs in 38 consecutive patients—28 males (73%) and 10 females (26%)—aged between 52 and 92 years (mean age: 72 ± 8.92 years). *Results*: Total clearance was recorded in 51% of lesions, while partial clearance was recorded in 73% of lesions. An excellent tolerability profile and high compliance rate were observed, with no treatment discontinuation due to the onset of adverse events. *Conclusion:* Our real-life experience confirms the effectiveness and safety of tirbanibulin ointment for the treatment of AKs.

## 1. Introduction

Actinic keratosis (AK) is a neoplasm of intraepithelial keratinocytes commonly found in sun-exposed areas in adults, especially the face, scalp and dorsal hands [1]. Risk factors include older age, male gender, Fitzpatrick skin types I–II, chronic ultraviolet (UV) radiation exposure, sunbed use, prolonged immunosuppression and prior history of AKs and non-melanoma skin cancer (NMSK) [2,3]. Olsen’s clinical grading categorizes AK lesions as grade 1 (faint pink macules that are barely visible but easily felt), grade 2 (moderately thick, red and scaly lesions that are easily felt and seen) and grade 3 (very thick, hyperkeratotic lesions that are sometimes challenging to differentiate from early cutaneous squamous cell carcinomas [cSCCs]) [4]. However, recent studies highlight discrepancies between clinical and histological grading, with Olsen grading lower than the histological grade in 62.4% of cases [5]. It has been suggested that AK, even in its early stages, can progress directly to invasive cSCC, the so-called “differentiated pathway,” without following conventional stages of keratinocyte intraepidermal neoplasia, namely the “classical pathway” [6]. The documented risk of progression to cSCC varies, with estimates ranging from less than 0.025% to 16% per lesion per year [7]. Due to the unpredictable course of progression, it is recommended to treat all AKs, regardless of their clinical grade [6]. To date, several topical treatments are licensed for AK treatment, including 4% 5-fluorouracil (5-FU) cream, 0.5% 5-FU plus 10% salicylic acid (5-FU/SA), 3% diclofenac sodium in hyaluronic acid (HA) gel and 3.75% and 5% imiquimod (IMI) cream, with different schedules of application that should be taken into consideration on the basis of patients and disease characteristics. Many of them are often limited by the onset of local skin reactions (LSRs), prolonged treatment duration, poor tolerability and reduced patient adherence [8]. Tirbanibulin 1% ointment (Klisyri^®^, Almirall, S.A., Barcelona, Spain), a new topical field therapy approved for treating AKs on the face and scalp in adults, seems to reduce these issues because of its good efficacy in front of the short duration (only five consecutive days) and the mild or absent LSRs, as evidenced in the phase 3 trials [9]. Tirbanibulin induces cell cycle arrest and apoptotic cell death in proliferating cells by directly binding to tubulin, leading to the disruption of microtubules. Additionally, it interferes with Src tyrosine kinase signaling. However, data from a real-world setting outside of clinical trials are lacking. Herein we report our experience in a real-life setting.

## 2. Materials and Methods

### 2.1. Inclusion and Exclusion Criteria (Study Population)

We performed a spontaneous open-label, prospective non-randomized study to assess the effectiveness and safety of tirbanibulin 1% ointment for the treatment of AKs in a real-life setting. Between February 2023 and October 2023, patients diagnosed with multiple AKs were recruited by the dermatology unit of the University Hospital of Messina, Italy. Eligible participants were 18 years or older and clinically diagnosed with AKs localized on the face and scalp. Exclusion criteria for enrollment were pregnant or lactating women, known allergies to any component of the study drug, history of photosensitivity, active infections, immunosuppression and prior therapies with other topical treatment for AKs, including 5-FU 4% cream, IMI cream at concentrations of 5% or 3.75%, diclofenac sodium in HA gel, PDT, cryotherapy or surgical excision within 12 weeks. The procedures followed here were in accordance with the ethical standards of the responsible committee on human experimentation and with the Helsinki Declaration of 1975, as revised in 1983. We have not used patients’ names, initials or hospital numbers. All patients gave written informed consent prior to their participation, and written consent was obtained to publish images.

### 2.2. Study Design 

#### 2.2.1. Baseline Evaluation

Three face-to-face visits were scheduled. At baseline (T0), a physical examination of patients with the acquisition of clinical and dermoscopic images was performed. Clinically collected data included age, gender, phototype, personal history of skin cancer and previous treatments for AKs (concluded at least 12 weeks before). Location and clinical grade of the lesions according to Olsen classification were collected as well (Table 1). 

#### 2.2.2. Treatment Received by Patients

All patients received treatment with tirbanibulin 1% ointment once daily for five consecutive days. The ointment was to be applied once a day in sufficient quantities to cover the affected AK area with a thin layer, avoiding application on open wounds or injured skin as indicated in the package leaflet. 

#### 2.2.3. First Evaluation Post-Treatment (T1)

Patients were reassessed either in person or through the submission of digital photos when unable to reach the hospital between the third and seventh day after the completion of the therapy to evaluate LSRs. LSRs were defined as erythema, scaling, crusting, swelling, vesiculation or pustulation as well as erosions. The assessment of local reactions was carried out with the use of a semi-quantitative 4-point scale with scores of 0—absent, 1—mild (slightly or barely perceptible), 2—moderate (distinct presence) and 3—severe (marked or intense).

#### 2.2.4. Second Evaluation Post-Treatment (T2)

Clinical and dermoscopic pictures were recorded eight weeks (T2) after the start of the treatment to evaluate its efficacy. Efficacy was evaluated as total clearance (total disappearance of lesions) or partial clearance (75% reduction of lesions). The efficacy was also assessed according to the Olsen clinical grade. All patients were invited to rate their compliance with the treatment on a scale from 1 to 4 (1= poor, 2= moderate, 3= good, 4= excellent).

#### 2.2.5. Objectives and Endpoints

The study aimed to evaluate the clinical efficacy of tirbanibulin 1% ointment for AK treatment and the potential differences in therapeutic response with respect to Olsen grading.

The primary efficacy outcome was the percentage of lesions with complete (100%) clearance, while the second efficacy outcome was the percentage of lesions with partial clearance, defined as a reduction of at least 75% of clinical and dermoscopic patterns.

Secondary objectives included assessing side effects (type and severity) and patients’ adherence to the treatment.

#### 2.2.6. Statistical Analysis

Results were expressed as mean ± standard deviation (continuous variables) or absolute frequency and percentage (categorical variables). For statistical analysis of data, the two-tailed Pearson chi-square test was employed to compare categorical variables. A *p*-value< 0.05 was considered statistically significant.

## 3. Results

### 3.1. Patients Characteristics

From February 2023 to October 2023, 38 patients were enrolled—28 males (73%) and 10 females (26%) aged between 52 and 92 years (mean age: 72 ± 8.92 years). A total of 228 lesions were treated (173 (76%) in the face and 55 (24%) in the scalp). Lesions Olsen’s grade 1 has been detected in 37% of lesions, grade 2 in 51% of lesions and grade 3 in 12% of lesions. All patients had undergone one or more previous treatments: 57% with PDT, 31% with cryotherapy, 15% with 3% diclofenac in HA gel and 10% with 4% 5-FU cream and with 3.75% IMI cream. 

Personal history of melanoma was recorded in 5% of patients, while 21% and 10% have been affected by SCC and BCC, respectively. Patients’ characteristics are summarized in Table 1.

### 3.2. Efficacy

All patients completed the study. At T2, total clearance (100%) was recorded in 51% of lesions, while partial clearance (reduction >75%) was recorded in 73% of lesions. According to the clinical grade of AK lesions, total clearance was observed, respectively, as follows: grade 1—51/85 lesions (60%), grade 2—57/116 lesions (49%) and grade 3—8/27 lesions (29%). A significantly higher prevalence of complete responses were observed in grade 1 group compared to grade 3 group (60% vs. 29%; *p* = 0.01), whereas no significant differences were observed between the other groups (grade 1 vs. grade 2 and grade 2 vs. grade 3). Partial clearance was observed as follows: grade 1—67/85 lesions (78%), grade 2—84/116 lesions (72%) and grade 3—15/27 lesions (55%). A significantly higher prevalence of complete responses were observed in grade 1 group compared to grade 3 group (78% vs. 55%; *p* = 0.02), while no significant differences were observed between the other groups (grade 1 vs. grade 2 and grade 2 vs. grade 3) (Table 2). 

**Figure 1:**
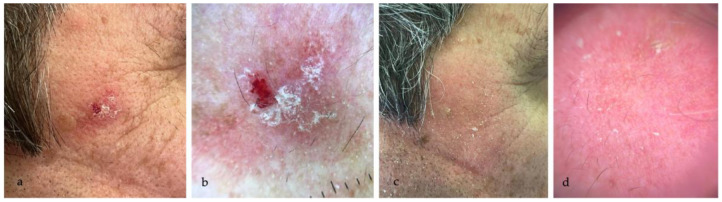
(**a**) AK in the right temporal area in a 78 year-old man; (**b**) dermoscopic examination (10×) highlights Olsen 3 AK, with yellowish scales and erosion; (**c**) complete resolution of AK, without sign of LSRs at eight weeks follow-up; (**d**) dermoscopy (10×) confirms the disappearance of AK, with complete resolution of the dermoscopic typical patterns.

**Figure 2:**
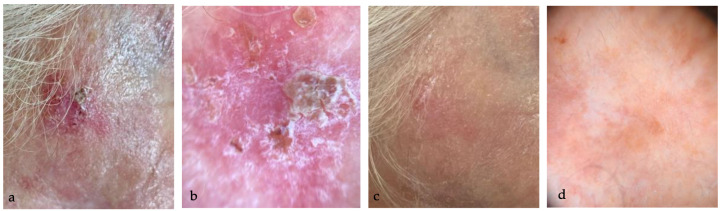
(**a**) AK in the right temporal area of a 79 year-old woman; (**b**) dermoscopy (10×) highlights Olsen 3 AK with scales and erosion; (**c**) complete resolution of AK, without sign of LRSs at eight weeks follow-up; (**d**) dermoscopy (10×) confirms the complete disappearance of AK typical patterns, with only slight hypopigmented halo.

**Figure 3:**
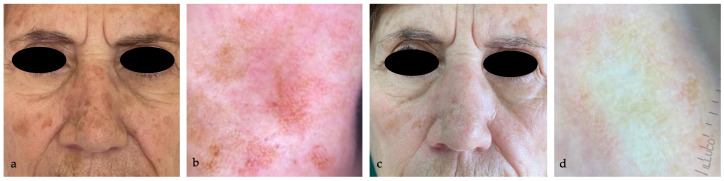
(**a**) AKs in the nose of an 80 year-old woman; (**b**) dermoscopy (10×) highlights Olsen 2 AK with rosettes; (**c**) complete resolution of AK in all the treated areas, without sign of LRSs at eight weeks follow-up; (**d**) dermoscopy (10×) confirms the complete disappearance of AK typical patterns.

**Figure 4:**
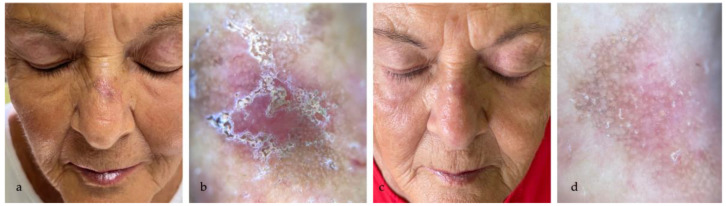
(**a**) AK in the nasal bridge in a 72 year-old woman; (**b**) dermoscopy (10×) highlights Olsen 3 AK, with scales and erosion; (**c**) clinical partial resolution of AK at eightweeks follow-up; (**d**) reduction of the AK dermoscopic patterns, with persistence of perifollicular pseudo-network.

The treatment efficacy was highlighted by the dermoscopic disappearance or reduction of the typical AK dermoscopic patterns (perifollicular red pseudo-network pattern, yellowish scales, keratotic follicular openings), as shown in Figure 1, Figure 2, Figure 3 and Figure 4.

### 3.3. Local Adverse Events

**Figure 5:**
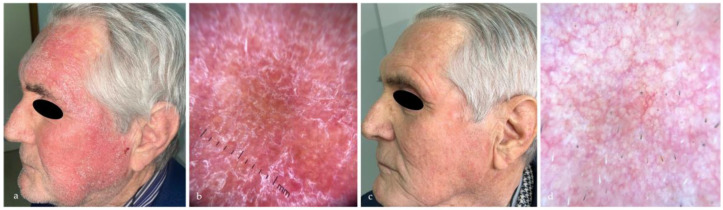
(**a**) Severe erythema and desquamation in the left cheek and frontal region in an 81 year-old man, recorded at T1; (**b**) dermoscopic examination (10×) reveals vascular pattern and desquamation in the treated areas; (**c**) complete resolution of the erythema and desquamation two months after starting the treatment; (**d**) dermoscopic examination (10×) reveals complete resolution of the dermoscopic inflammatory patterns two months after the treatment.

LSRs were evaluated at T1. Moderate erythema was the most frequent LSR recorded in 23/38 patients (60%), followed by moderate desquamation in 17/38 patients (44%). No severe crusting, swelling, pustulation and erosion was reported. LSRs typically appeared on day 7–9 after starting the treatment and peaked on day 10-12, before spontaneously resolving in about 2–4 weeks. LSRs are summarized in Table 3. Figure 5 represents an example of severe erythema, scaling and moderate swelling recorded at T1, with complete resolution at one month.

### 3.4. Patient Compliance

Treatment was generally well tolerated by all patients. No patients discontinued the therapy due to early- or late-onset of LSRs. 

71% of patients rated their compliance with the treatment as 4 (excellent), 18% as 3 (good) and 8% as 2 (moderate). Only one patient rated their compliance as poor (Table 4). 

## 4. Discussion

AKs are cutaneous intraepithelial neoplasms typically localized on chronically sun-damaged skin, affecting approximately 60% of predisposed individuals over the age of 40 [1,10]. Several treatments with different efficacy and safety profiles are currently available for AK management [11]. Therapies classified as “lesion directed” are performed on a single or very few AK lesions within the same area, while therapies that are “field directed” target the cancerization field as a whole [10]. Single or few discrete AKs are typically treated with cryotherapy or ablative lasers, while multiple lesions and surrounding photo-damaged skin—the cancerization field—usually require the use of topical agents or PDT [12]. In daily practice, a blend of treatment modalities are frequently used to enhance clearance rates, especially in field cancerization. Field therapies are often associated with LSRs and must be administered over periods of weeks or months, which may reduce patients’ adherence and compromise treatment success. In this context, tirbanibulin ointment is demonstrating good efficacy, an adequate safety profile and excellent patient adherence. Tirbanibulin is a novel synthetic anti-proliferative agent that inhibits tubulin polymerization, a structural protein involved in cell migration, protein transport and cell division. It also promotes the induction of p53 and cell cycle arrest at the growth 2 and mitosis (G2/M) interphase of proliferating cells and activates the intrinsic and extrinsic apoptosis signaling cascade via hyperphosphorylation of Bcl-2, cleavage of caspases 8 and 9, activation of caspases 3 and poly (ADP-ribose) polymerase cleavage [13]. Tirbanibulin also indirectly downregulates Src kinase signaling, which is increased in AKs and cSCC, through the disruption of the microtubule network which interferes with the cell signaling pathways that regulate Src expression. Tirbanibulin efficacy was evaluated in two crucial phase 3 trials, reaching complete clearance in 44–54% of patients and in 76–82% of lesions [9]. Such results have found confirmation in our real-life evaluation, with half of the lesions achieving complete clearance and almost two-thirds reaching partial clearance. Moreover, a statistically significant higher complete clearance rate was observed at the eight-week follow-up in Olsen grade 1 lesions compared to Olsen grade 3 lesions (60% vs. 29%; *p* = 0.01). A higher partial response rate was also observed at the eight-week follow-up in the grade 1 group compared to the grade 3 group (78% vs. 55%; *p* = 0.02), thus suggesting that tirbanibulin performs better on thin lesions, as expected. To date, poor real-life data are available, with heterogeneity in the methods of efficacy evaluation and heterogeneity in the results, making them difficult to compare [14,15,16]. However, the data on tiranibulin’s safety profile agrees, with excellent tolerability reported [14,15,16]. In fact, compared to other topical treatments already on the market for AKs, tirbanibulin is showing many advantages in terms of safety and patient adherence [17]. Current first-line topical therapies for AKs, including 4% 5-FU cream, 5-FU/SA, 3% diclofenac sodium in HA gel and 3.75% and 5% IMI cream, although performing very well, present several limitations that undermine compliance and, consequently, therapeutic success [12]. Topical 4% 5-FU cream is a thymidylate synthase inhibitor used to rapidly induce apoptosis of dividing cells. Its efficacy has been evaluated in a randomized double-blind phase 3 trial, with complete clearance response (CCR) and partial clearance response (PCR) of 24% and 74%, respectively, achieved after a treatment period of four consecutive weeks of daily application [18]. Data from real-life study investigating safety and efficacy of 4% 5-FU plus emollient versus 4% 5-FU alone, evidenced a 33.3% of CCR and 55.1% of PCR in the group treated by 4% 5-FU alone. Local irritation remains the primary source of harm to patients and often the primary reason for the discontinuation of treatment, not influenced by the addition of emollient cream. The mean LSR total scores at week four showed no statistically significant differences between patients who received 4% 5-FU plus emollient and patients who received 4% 5-FU alone [19]. The 5-FU/SA cream has been evaluated for the treatment of slightly palpable and moderately thick hyperkeratotic AK lesions in a randomized controlled trial with a CCR of 49.5% reached eight weeks after the end of the twelve-week treatment. Major limitations were the onset of LSRs, which correlated with treatment duration and efficacy [20]. A network meta-analysis compared the efficacy of 5-FU with other treatments and highlighted that 5-FU cream and 5-FU/SA were more effective, with a CCR of 56.8% and 35.7%, respectively. The 5-FU/HA cream also performed better than diclofenac 3% in HA in a randomized, placebo-controlled, double-blind, parallel-group multicenter trial [21].

Diclofenac 3% in HA is a nonsteroidal anti-inflammatory drug (NSAID) that has a greater affinity for the inducible form of cyclooxygenase (COX-2) than the constitutive (COX-1) form of this enzyme. Although the precise mode of action remains to be fully elucidated, diclofenac 3% in HA has the potential to enhance the delivery of diclofenac to sites of inflammation and/or neoplasia, which play anti-inflammatory, anti-proliferative and pro-apoptotic effects. The major point of strength is the good profile of safety being generally well-tolerated owing to its few side effects. However, the efficacy is limited, especially in thick lesions. Pooled data from two vehicle-controlled studies of twice daily ninety-day treatments with diclofenac showed CCR in 42% of treated patients compared to 14% of vehicle-treated participants, with CCR at one year between 18% and 29% [22,23]. Adherence to the therapy may be compromised by the prolonged duration of the treatment, with twice daily application of the drug up to three months. Even in patients following a low-intensity intervention program including periodic visits, a brief health education intervention, enhanced patient-physician communication and a weekly SMS reminder to medication prescriptions, there were no significant differences versus standard of care regimen in terms of treatment adherence [24]. As with other oral and topical medications in the class, NSAIDs carry a black box warning for cardiovascular and gastrointestinal side effects [11].

IMI cream is a topical immunomodulator that acts through the activation of toll-like receptor 7 (TLR7), which in turn triggers a cascade of events that lead to the production of various cytokines, particularly interferon-alpha (IFN-α) and tumor necrosis factor-alpha (TNF-α). This increased immune activity helps to eliminate abnormal or damaged cells, including the pre-cancerous cells associated with actinic keratosis [11]. Topical application of IMI cream in various concentrations showed moderate to large benefits for the management of AK in various anatomic locations. Four placebo-controlled studies provided data on the efficacy of 3.75% IMI cream for the treatment of AKs on the face and scalp with a complete clearance rate between 34% and 59.5% [25].

PDT is a field-therapy, non-invasive photochemotherapeutic procedure based on the topical application of 5-aminolaevulinic acid (ALA) or its methylated ester (MAL), two light-responsive prodrugs, which, after irradiation with a red light of ~630nm, stimulate cytotoxic reactive oxygen species, causing selective destruction of cells with a high metabolic state, including cancerous and pre-cancerous ones [26,27]. Pooled data from three studies on up to two ALA-red light PDT treatments showed rates of baseline lesion clearance of 89.1% and 32.7% at twelve weeks post-treatment for ALA-PDT and placebo-PDT patients, respectively [28,29,30]. 

Currently, the choice of treatment for AKs often depends on the preferences of patients and their treating physicians. Evidence from randomized trials with direct comparison between treatments and with long-term follow-up is scarce. A multicenter, single-blind, controlled trial compared treatment success at 12 months of 5% 5-FU cream, 5% IMI cream and MAL-PDT in patients with AK lesions of any grade. The cumulative probability of treatment success for 5-FU was 74.7%, and for IMI and MAL-PDT it was 53.9% and 37.7%, respectively [31]. Well-conducted randomized controlled trials (RCTs), systematic reviews and meta-analyses tried to assess AK treatments and their combinations [32,33,34,35,36]. Existing meta-analyses consistently indicate that 5-FU formulations and PDT are the most effective options for AK reduction, regardless of their location. However, the majority of RCTs, meta-analyses and guidelines primarily focus on short-term outcomes assessed 8–12 weeks to approximately 6 months after the completion of treatment [33,35,37]. While these evaluations capture short-term results such as lesion clearance, they do not provide insights into the true long-term outcomes of the considered interventions. It is noteworthy that regulatory agencies often approve AK treatments based on short-term outcomes like clearance after eight weeks or transient side effects, even though the more significant medical concerns involve long-term clearance and irreversible side effects [38]. As for the new arrival, tirbanibulin, a recent network meta-analysis of data from RCTs demonstrated that the efficacy and safety profile of tirbanibulin is in line with existing topical therapies for AK already approved in Europe [17]. However, to the best of our knowledge, there are no current head-to-head studies available comparing the efficacy of tirbanibulin with other therapies, perhaps due to its recent market introduction. Such studies would be valuable in determining the most effective topical therapy.

Moving to adverse events, topical therapies frequently cause several moderate to severe LSRs (erythema, flaking, erosions, ulcerations and irreversible skin changes of pigmentation and scarring) with prolonged patient discomfort, sometimes requiring the temporary suspension or withdrawal of the treatment [8]. Moreover, the severity of LSRs is often unpredictable, varying from mild irritation to severe vesiculation until bleeding erosion [39]. In addition, they require a long period of application ranging from four weeks up to twelve weeks with one or two daily applications [40,41]. Long-lasting AK treatment can be quite distressing for patients, as it can interfere with daily activities such as work and social engagements, especially for lesions localized in aesthetically sensitive areas such as the face and scalp. Conversely, treatment adherence and patient satisfaction with tirbanibulin therapy have been significantly better than other available therapies, probably because of its short period of application [17]. Our experience confirms the high percentage of patients satisfied in a real-life setting. Tirbanibulin’s safety profile with milder LSRs reflects its mechanism of action. It causes cell death by apoptosis and not by necrosis, leading to a reduction of proinflammatory cytokine release [13,42]. These findings are confirmed by our study. Although many patients experienced mild or moderate erythema and scaling, they were self-resolving, well-tolerated by the patient and did not require early discontinuation of the treatment. Furthermore, adherence to therapy is improved due to the short duration of only five days of application. However, our study presents some limitations: the limited number of patients, necessitating a real-life experience on a larger sample and the short follow-up period. It would be beneficial to revisit these patients after one year to observe the percentage that have maintained the outcome and the percentage with disease recurrence. Moreover, the lack of a control group can pose difficulties in establishing a direct comparison between the group receiving treatment and one not receiving any treatment. This challenge compromises the ability to conclusively attribute the observed outcomes specifically to the intervention being studied.

Finally, owing to its high tolerability, the use of tirbanibulin could be considered for the simultaneous treatment of several areas of 25cm^2^ to treat a larger field of cancerization simultaneously. Furthermore, to treat more hyperkeratotic lesions (Olsen grade 3), a possible pre-treatment with salicylic acid or keratoregulators derived from vitamin D could be evaluated to reduce the thickness of the keratotic component, thus ensuring that tirbanibulin can carry out its therapeutic effect [43]. 

## 5. Conclusions

Tirbanibulin 1% ointment is a new topical therapy approved for the treatment of AKs of the face and scalp, whose effectiveness has been demonstrated in registry studies. Our real-life experience confirms the effectiveness and safety of tirbanibulin, with a complete response rate of 51% and partial response rate of 73%. No patients interrupted the therapy for the onset of adverse events or LSRs. Compared to other currently available topical therapies, tirbanibulin performs with comparable efficacy, ensuring a good safety profile with milder side effects and reducing rates of early therapy discontinuation or loss of adherence. One of the major points of strength seem to be the short-term schedule of application, evidenced by the patients’ excellent compliance rate. Our real-life study confirms that tirbanibulin represents a manageable and safe tool, with a good efficacy profile for the treatment of AKs of the face and scalp. It remains to evaluate and compare the long-term efficacy and safety after one year, and to validate its use on larger surfaces to 25 cm^2^ and on other anatomical areas like upper-chest and arms.

## Figures and Tables

**Table 1:** Patients’ characteristics.

Median Age (years)	72 (±8.92)
**Sex (%)**	
Male	28 (73%)
Female	10 (26%)
**Fitzpatrick skin type (%)**	
I	0 (0%)
II	10 (26%)
III	28 (73%)
**AK location (%)**	
Scalp	173 (76%)
Face	55 (24%)
**AK clinical grade (%)**	
Olsen 1	85 (37%)
Olsen 2	116 (51%)
Olsen 3	27 (12%)
**Previous treatments (%)**	
Cryotherapy	31%
Diclofenac in HA	15%
5-FU 4%	10%
IMI 3,75%	10%
PDT	57%
None	31%
**History of skin cancer (%)**	
Melanoma	5%
cSCC	21%
BCC	10%
No history	64%

**Table 2:** Tirbanibulin efficacy according to Olsen’s clinical grade.

Olsen Grade	N° of Lesions (%)	Complete Response no./Total no. (%)	Partial Response No./Total No. (%)
**Grade 1**	85 (37%)	51/85 (60%) *	67/85 (78%) *
**Grade 2**	116 (51%)	57/116 (49%)	84/116 (72%)
**Grade 3**	27 (12%)	8/27 (29%)	15/27 (55%)
**All grades**	228	116/228 (51%)	166/228 (73%)

* Grade 1 vs. grade 3; *p* < 0.05.

**Table 3:** LSRs recorded at T1.

	Mild	Moderate	Severe
**Erythema**	9/38 (24%)	23/38 (60%)	4/38 (10%)
**Scaling**	9/38 (24%)	17/38 (44%)	3/38 (8%)
**Crusting**	8/38 (21%)	6/38 (15%)	-
**Swelling**	7/38 (18%)	3/38 (8%)	-
**Vesiculation/** **Pustulation**	3/38 (8%)	1/38 (3%)	-
**Erosion/Ulceration**	2/38 (5%)	-	-

**Table 4:** Patient compliance, rated as excellent, good, moderate and poor.

	Patient Compliance Rate n (%)
**Excellent**	28 (71%)
**Good**	7 (18%)
**Moderate**	3 (8%)
**Poor**	1 (3%)

## Data Availability

All data generated or analyzed during this study are included in this article.
